# Preclinical analyses of intravesical chemotherapy for prevention of bladder cancer progression

**DOI:** 10.18632/oncotarget.852

**Published:** 2013-02-25

**Authors:** Joan C. Delto, Takashi Kobayashi, Mitchell Benson, James McKiernan, Cory Abate-Shen

**Affiliations:** ^1^ Department of Urology, Columbia University Medical Center, New York, NY, USA; ^2^ Department of Pathology and Cell Biology, Columbia University Medical Center, New York, NY, USA; ^3^ Herbert Irving Comprehensive Cancer Center, Columbia University Medical Center, New York, NY, USA

**Keywords:** Non-muscle invasive bladder cancer, intravesical therapy, genetically engineered mouse models, preclinical studies

## Abstract

There is a critical need to identify treatment options for patients at high risk for developing muscle invasive bladder cancer that avoid surgical removal of the bladder (cystectomy). In the current study, we have performed preclinical studies to investigate the efficacy of intravesical delivery of chemotherapy for preventing progression of bladder cancer. We evaluated three chemotherapy agents, namely cisplatin, gemcitabine, and docetaxel, which are currently in use clinically for systemic treatment of muscle invasive bladder cancer and/or have been evaluated for intravesical therapy. These preclinical studies were done using a genetically-engineered mouse (GEM) model that progresses from carcinoma *in situ* (CIS) to invasive, metastatic bladder cancer. We performed intravesical treatment in this GEM model using cisplatin, gemcitabine, and/or docetaxel, alone or by combining two agents, and evaluated whether such treatments inhibited progression to invasive, metastatic bladder cancer. Of the three single agents tested, gemcitabine was most effective for preventing progression to invasive disease, as assessed by several relevant endpoints. However, the combinations of two agents, and particularly those including gemcitabine, were more effective for reducing both tumor and metastatic burden. Our findings suggest combination intravesical chemotherapy may provide a viable bladder-sparing treatment alternative for patients at high risk for developing invasive bladder cancer, which can be evaluated in appropriate clinical trials.

## INTRODUCTION

Bladder cancer is the fourth most common cancer in men and the eighth most common overall, with 74,000 new cases diagnosed and an estimated 15,000 deaths in 2012 (1). Notably, distinct subtypes of bladder cancer have very different patient outcomes (2-5). In particular, the lethal form is muscle-invasive disease, for which the precursor is carcinoma *in situ* (CIS) (6). The primary treatment for muscle invasive bladder cancer is cystectomy (surgical removal of the bladder), which is associated with significant morbidity; moreover, progression to metastatic disease has a particularly low 5-year survival (6-9). At the other end of the spectrum is non-invasive bladder cancer, which presents as papillary lesions and generally has good patient prognosis (6, 9). However, non-muscle invasive bladder cancer can progress to a high-risk disease that ultimately gives rise to muscle invasive bladder cancer.

Many patients with recurrent non-muscle invasive bladder cancer are treated with intravesical delivery of Bacillus Calmette Guerin (BCG), an immunotherapy regime (10). Although widely used, there can be significant adverse reactions to BCG; moreover, 30% of patients do not respond and even those that respond have a 20% chance of progression (10, 11). For patients with high-risk recurrent non-muscle invasive bladder cancer, including those who have failed BCG therapy, early cystectomy with urinary diversion is currently the preferred treatment option (10). However, cystectomy is associated with significant morbidity, which severely impacts quality of life, and it may not be a viable option for patients who are medically unfit for surgery. Importantly, patients who undergo early cystectomy before they progress to invasive bladder cancer may result in overtreatment.

Thus, there is an urgent need to identify alternative, bladder-sparing therapies for patients with high-risk non-muscle invasive bladder cancer. One such option is intravesical delivery of chemotherapy to prevent progression to invasive bladder cancer. For example, our phase I clinical trial using intravesical docetaxel yielded a 56% complete response rate with 22% durability of response in three years (12, 13). Other clinical trials have shown that gemcitabine has promising results for patients with recurrent non-muscle invasive bladder cancer in Phase I and Phase II clinical trials (14-16). Intravesical delivery of selected agents has also been investigated preclinically in Xenograft models based on orthotopic implantation of human bladder cancer cells into immunodeficient mouse hosts (17, 18). Although these clinical and preclinical studies using intravesical chemotherapy are promising, systemic chemotherapy, administration of single agents has rarely resulted in durable long-term remissions. In fact, the most successful systemic chemotherapy for advanced metastatic bladder cancer is either a two-drug regimen of gemcitabine and cisplatin or a four-drug combination regimen of methotrexate, vinblastine, adriamycin and cisplatin (MVAC) (8, 9, 19, 20).

We, therefore, reasoned that the design of optimal intravesical chemotherapy regime(s) for patients with recurrent non-muscle invasive bladder cancer would benefit from preclinical studies aimed at a direct, side-by-side comparison of drug regimens involving single versus double combinations. Toward this end, we have now performed preclinical studies using a genetically engineered mouse (GEM) model of progressive bladder cancer to systematically analyze the efficacy of chemotherapeutic agents when delivered individually versus in combination. Our preclinical studies utilize a GEM model of progressive bladder cancer based on bladder-specific deletion of two tumor suppressor genes, *p53* and *Pten*, which are frequently de-regulated in invasive bladder cancer (21). Following tumor induction by delivery of Adeno-Cre into the bladder lumen, these *p53*^*f/f/*^; *Pten*^*f/f*^ mice develop carcinoma *in situ* (CIS) by 8 weeks, which progresses to invasive bladder cancer with prevalent metastases by 5 months of age (21, 22). The bladder tumors from these Adeno-Cre infected *p53*^*f/f/*^; *Pten*^*f/f*^ mice display similar histologic features as human muscle invasive bladder cancer, and their metastases arise in similar tissues, as occurs in humans (21). Our previous analyses of this GEM model have provided molecular insights regarding bladder cancer progression and this model has also provided an effective resource for *in vivo* preclinical studies (21, 22). Indeed, we have demonstrated that bladder tumors arising in these Adeno-Cre infected *p53*^*f/f/*^; *Pten*^*f/f*^ mice respond to systemic treatment with rapamycin, while intravesical delivery of rapamycin prevents progression of CIS to invasive bladder cancer (21, 22).

In the current study, we have performed preclinical studies using this GEM model to evaluate the efficacy of intravesical delivery of chemotherapy for prevention of progression to invasive bladder cancer. We evaluated three chemotherapy agents, namely cisplatin, gemcitabine, and docetaxel, which are standardly administered systemically for treatment of invasive, metastatic bladder cancer (8, 9, 19, 20). We directly compared these agents to assess their relative efficacy when delivered individually or in combination for prevention of progression to invasive bladder cancer. When tested individually, intravesical delivery of gemcitabine is most effective among the three agents for delaying progression to muscle-invasive bladder cancer; however, each combination of two agents was more effective for reducing both tumor and metastatic burden. These findings suggest that patients at high-risk for developing invasive bladder cancer may be candidates for combination intravesical chemotherapy.

## RESULTS

**Figure 1:**
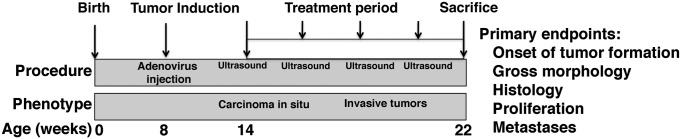
Study design for preclinical analyses of intravesical chemotherapy Tumor induction was initiated by delivery of Adeno-Cre directly into the bladder lumen of *p53*^*f/f/*^; *Pten*^*f/f*^ female mice at 8 weeks. Six weeks later (at 14 weeks), mice were imaged using ultrasound (baseline) and then enrolled into one of 8 treatment arms for the preclinical intravesical treatment: vehicle; gemcitabine, docetaxel or cisplatin as single agents, or cisplatin + docetaxel, gemcitabine + docetaxel, or gemcitabine + cisplatin in combination. The treatments were continued for 8 weeks during which time the bladders were imaged every two weeks using ultrasound. At the conclusion of the treatment period (22 weeks) mice were sacrificed for analyses including the endpoints indicated.

We performed preclinical studies to evaluate the consequences of intravesical chemotherapy for delaying progression from CIS to invasive bladder cancer using a genetically-engineered mouse model that recapitulates these progression stages. In particular, we initiated preclinical treatment in *p53*^*f/f/*^; *Pten*^*f/f*^ mice six weeks after tumor induction with Adeno-Cre (*i.e.*, at 14 weeks of age; Fig. 1), since, as we have shown previously, by this time-point these GEM mice have developed CIS, but have not progressed to invasive bladder cancer (22). Prior to initiation of treatment, we confirmed the absence of overt tumors using ultrasound imaging. Cohorts of mice were then randomly assigned to the various treatment arms, which were the Vehicle group, the single agent group (cisplatin, gemcitabine, or docetaxel), and the combination group (cisplatin + gemcitabine; gemcitabine + docetaxel; and cisplatin + docetaxel) (Fig. 1). In initial pilot studies, we found that the mice were able to tolerate each of these agents via intravesical delivery, and were able to tolerate up to two doses of intravesical treatment weekly (data not shown). Therefore, we performed these preclinical studies by combining a maximum of two agents for a biweekly instillation. Each of the single agent and double combination treatment groups displayed no significant weight loss, based on bi-weekly measurements, nor did they display other overt signs of distress.

Cohorts of mice were treated one time weekly (for the signal agents) or bi-weekly (for the double combinations) for a period of 8 weeks (Fig. 1). By this point following tumor induction (*i.e.*, at 22 weeks), the vehicle-treated Adeno-Cre-infected *p53*^*f/f/*^; *Pten*^*f/f*^ mice characteristically develop invasive bladder tumors that are large in size, have histological features of invasive bladder cancer, including a high rate of proliferation, and are highly metastatic (21). Therefore, our analyses focused on whether intravesical treatment with the individual or combination chemotherapy regimes prevented progression to invasive bladder.

**Figure 2:**
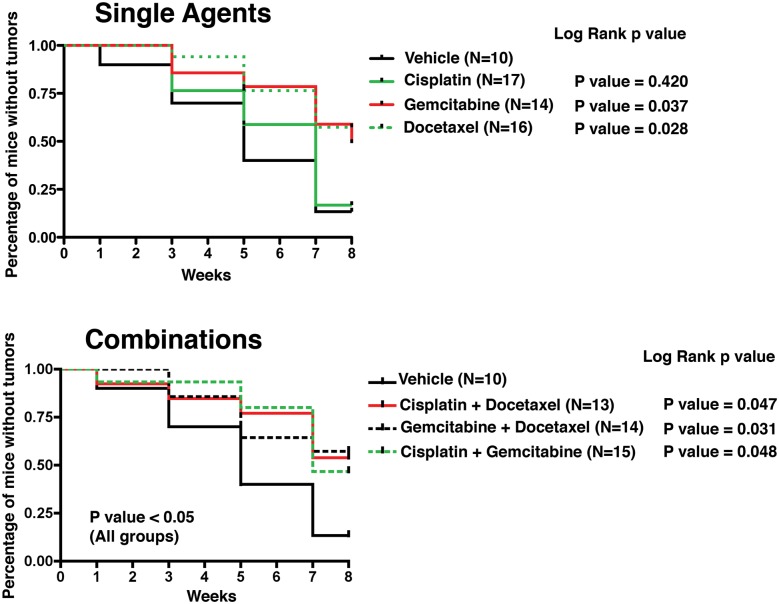
Onset of tumor formation detected by ultrasound imaging Graphic representation of the percentage of mice tumor-free over the course of treatment as detected by ultrasound imaging showing the single (top) and combination (bottom) images. The log rank p-values are indicated for each treatment (compared to Vehicle). Pictures of the ultrasound images are shown in Figure 4.

Following treatment initiation, the experimental mice were monitored with ultrasound every two weeks for detection of tumors (Fig. 1, Fig. 2). During the 8-week treatment period, the vehicle-treated mice rapidly developed tumors as detected by ultrasound imaging, as expected (Fig. 2). In contrast, mice treated with gemcitabine or docetaxel, but not cisplatin, as single agents developed tumors at a slower rate (Fig. 2). Moreover, mice that received each of the combined treatments (cisplatin + docetaxel; gemcitabine + docetaxel; and cisplatin + docetaxel) also displayed a significant delay in the formation of overt tumors (p < 0.05; Fig. 2). These findings indicate that intravesical chemotherapy with gemcitabine or docetaxel alone or with the three combinations of agents delays overt tumor formation in tumor-prone mice.

**Figure 3:**
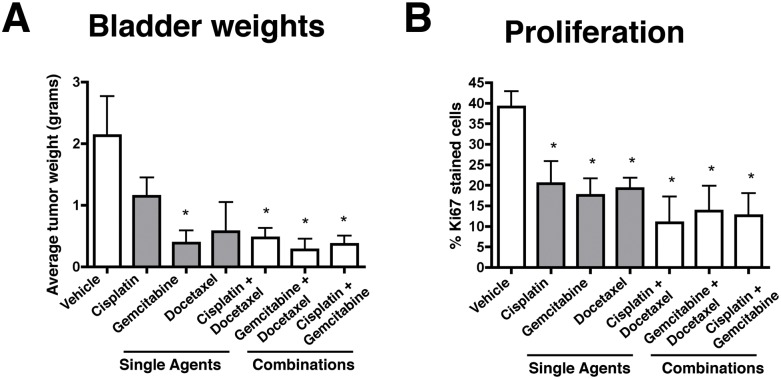
Endpoint analyses for preclinical studies Summary of data from experimental mice following treatment with Vehicle or with the single or combination agents as indicated. A) Summary of Bladder weights. B) Summary of cellular proliferation as evaluated by Ki67 immunostaining of the treated bladders.

**Figure 4:**
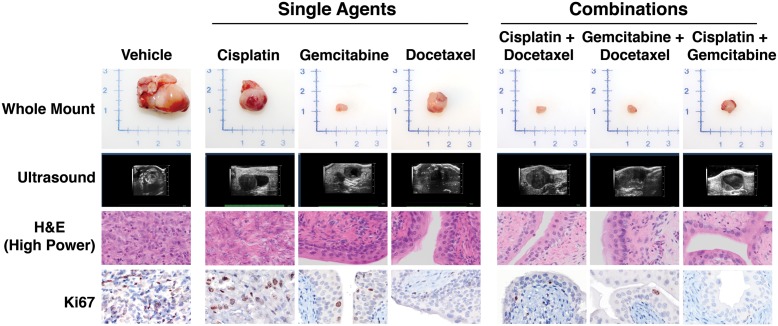
Phenotype of mice treated following intravesical treatment Shown are representative images of bladders from Adeno-Cre infected *p53*^*f/f/*^; *Pten*^*f/f*^ mice following treatment with Vehicle or with the single or combination agents as indicated. The *top* panels show whole mount images of the dissected bladder at the time of dissection. The *upper middle* panels show representative ultrasound images and representative H&E images of bladder histology at the conclusion of the treatment. The *bottom* panels show images of representative of Ki67-immunostaining for quantification of proliferation.

At the end of the 8-week treatment period, the cohorts of experimental mice were sacrificed and their bladders and other tissues were evaluated. As expected, all of the vehicle-treated mice displayed large bladder tumors (~2 grams) that were readily evident upon gross inspection (Table 1, Fig. 3A, 4; Supplementary Fig. 4). In contrast, mice in each of the treatment groups treated with intravesical chemotherapy displayed a reduction in the size of the bladder, although the extent differed, particularly for mice treated with the single agents (Table 1, Fig. 3A). In particular, the bladder weights of mice treated with gemcitabine alone were significantly reduced (5.4 fold reduced, *p* = 0.0271), whereas those treated with docetaxel or cisplatin were reduced in weight (1.8 and 3.73 fold reduced, respectively) the mean values were not significantly different compared to the Vehicle-treated mice (Table 1, Fig. 3A, 4, Supplementary Fig. 1). In combination, the reduction in bladder size was further augmented for gemcitabine in combination with either docetaxel (7.6 fold reduced, *p* = 0.0194) or cisplatin (6.0 fold reduced, *p* = 0.0250), and the combination of cisplatin + docetaxel also resulted in a significant reduction in bladder weight albeit to a lesser extent than the combinations with gemcitabine (4.53 fold; *p* = 0.0.0311; Table 1, Fig. 3A, 4, Supplementary Fig. 1). Notably, by gross inspection, bladders treated with the combination agents, and to a lesser extent gemcitabine alone, were similar in size and appearance to normal bladder (~0.20-0.30 grams), consistent with the interpretation that there was minimal tumor volume (Fig. 4, Supplementary Fig. 1). Therefore, intravesical treatment with either gemcitabine alone or with the various combinations of agents resulted in a significant reduction of tumor burden at the end of the treatment period.

We evaluated the histopathology of the bladders following intravesical chemotherapy by examining multiple H&E sections throughout the bladder using whole slide imaging (Table 1, Fig. 4). As evident in representative high power images (Fig. 4), the vehicle treated mice display invasive bladder cancer as has been described previously (21). The experimental mice treated with the single chemotherapy agents displayed varying degrees of histopathology that were consistent with their gross bladder phenotypes. In particular, the bladder epithelium of most of the mice treated with either gemcitabine or docetaxel was multilayered with areas of hyperproliferation, reminiscent of to CIS, but did not display evidence of invasion, whereas the epithelium of most of the mice treated with cisplatin alone, which had evident bladder tumors, displayed histological evidence of invasion (Table 1, Fig. 4). Moreover, the histology of the bladders of most of the experimental mice treated with each of the double combinations had some evidence of CIS, but were otherwise relatively normal in appearance with little or no evidence of invasion (Table 1, Fig. 4). These findings further support the efficacy of combination chemotherapy for prevention of bladder cancer progression.

**Table 1:** Summary of treatment endpoints

			Bladder Weight				Phenotype	Proliferation		Metastases	
	Treatment	N	Mean weight (gr)	SEM	Fold Change	P value	Description	% Ki67	P value	Cases w/ Mets	P value
Vehicle	PBS (DMSO in PBS)	22	2.13 (N = 10)	0.64	-	-	Large tumors (18/22); Small tumors (4/22)	39.1 (N = 4)	-	15/18 (85%)	-
Single Agents	Cisplatin	15	1.15 (N = 7)	0.30	1.85	NS	Small tumors (12/15); CIS (3/15)	20.4 (N = 4)	0.038	6/14 (42%)	NS
	Gemcitabine	14	0.39 (N = 5)	0.20	5.46	0.0271	Small tumors (3/14); CIS (11/14)	17.5 (N = 4)	0.019	4/10 (40%)	NS
	Docetaxel	14	0.57 (N = 5)	0.48	3.73	NS	Small tumors (5/14); CIS (11/14)	19.2 (N = 4)	0.013	4/10 (40%)	NS
Combinations	Cis + Doce	22	0.47 (N = 13)	0.16	4.53	0.0311	Small tumors (3/22); Normal/CIS (19/22)	10.9 (N = 4)	0.032	2/12 (16%)	0.004
	Gem + Doce	23	0.28 (N = 13)	0.18	7.60	0.0194	Small tumors (2/23); Normal/CIS (21/23)	13.7 (N = 4)	0.017	1/13 (8%)	0.001
	Cis + Gem	26	0.36 (N = 14)	0.14	6.00	0.0250	Small tumors (4/26); Normal/CIS (22/26)	12.6 (N = 4)	0.011	2/15 (13%)	0.003

As an additional parameter of disease progression, we evaluated the proliferation of the bladder epithelial and/or tumor cells of the experimental mice following treatment with intravesical chemotherapy. As we have shown previously (21), the vehicle treated mice display a high rate of proliferation (~40%) (Fig. 3B, Table 1). Consistent with the tumor size and histological phenotype, which was significantly abrogated by the intravesical chemotherapy, the single agents, and particularly gemcitabine and docetaxel, displayed a significant reduction in proliferation (17-20%; *p* = 0.012 to 0.04). Moreover, mice treated with the combination agents displayed a more profound reduction in proliferation (10.9% to 13.7%; *p* = 0.01 to 0.03). These findings further underscore the efficacy of combination intravesical chemotherapy for prevention of bladder cancer progression.

Finally, we evaluated whether treatment with intravesical chemotherapy affected the development of metastases. As we expected based on previous analyses (21), at the conclusion of the treatment period the majority of the vehicle-treated mice (15/18) had prominent overt metastases to several tissues including lymph nodes, liver, and pancreas (Table 1). The incidence of metastases was consistently reduced for the experimental mice treated with the single agents, although not significantly. However, mice treated with each of the combination chemotherapy regimes displayed a statistically significant reduction in metastases (2/12 to 2/15, *p* = 0.003 to 0.004; Table 1). Taken together, these findings demonstrate the efficacy of intravesical delivery of combination chemotherapy for prevention of progression to invasive, metastatic bladder cancer.

## DISCUSSION

Our study addresses the need to identify alternative, bladder-sparing treatment options for patients at risk for developing invasive bladder cancer. Toward this end, we investigated the efficacy of intravesical chemotherapy in a preclinical model of the disease. Several key aspects of our study advance the findings of previous clinical studies (12, 14), as well as preclinical analyses using an orthotopic xenograft model of invasive bladder cancer (18). First, we have used a GEM model that reliably exhibits progression from pre-invasive lesions to overt invasive disease, and therefore our study has enabled us to specifically address the efficacy of these treatments in the context of disease progression. Additionally, we have performed a direct, side-by-side comparison of intravesical delivery of three chemotherapy agents, namely cisplatin, gemcitabine, and docetaxel. This has enabled us to compare their efficacy when delivered alone or in combination. Lastly, we have evaluated multiple endpoints, which proved to be important when considering potentially clinically-relevant endpoints, such as the development of the metastases, in which the combination treatments outperformed any of the single agents.

The major conclusion of the current study is that while the single agents, and particularly gemcitabine, may be somewhat effective for reducing tumor burden, when considering all of the endpoints examined, the most effective regime for preventing progression to invasive bladder cancer is a combination agents, particularly those including gemcitabine. The further implication of our findings is that while intravesical treatment with gemcitabine, or with the other single agents, may have an immediate benefit for delaying progression, sustained effects that may impact overall survival, such as metastases, may require combination treatments. The additional implication of our study is that more than one combination is likely to be effective for preventing progression, which may leave open several options for patients who do not respond or tolerate one treatment regime but may benefit from an alternative.

We propose that our findings provide the rationale for evaluation of combination intravesical chemotherapy for patients with non-muscle invasive bladder cancer who are at high risk for progressing to invasive disease. Such treatment would be particularly effective for management of patients who are unable to undergo cystectomy, but may be extended to others who are also at high risk of progressing to muscle invasion and wish to avoid early removal of the bladder. Thus, combination intravesical chemotherapy may provide an alternative to cystectomy that improves the quality of life.

## MATERIALS AND METHODS

### Mouse model of progressive bladder cancer

All animal experiments were performed according to protocols approved by the Institutional Animal Care and Use Committee at Columbia University Medical Center. The genetically engineered mouse (GEM) model of progressive bladder cancer used in this study was developed and characterized in our laboratory and has been described previously (21). Briefly, these mice are based on bladder-specific deletion of floxed alleles of *p53* and *Pten* (*i.e.*, p53^*flox/flox*^; Pten^*flox/flox*^) in a C57/Bl6 strain background. Tumors are induced by delivery of an Adeno-virus expressing Cre recombinase (hereafter referred to as Adeno-Cre) directly into the bladder lumen at 8 weeks of age (21). Ultrasound imaging using a Vevo 2100® Imaging System (Visual Sonics, Toronto, Ontario, Canada) was performed to detect bladder tumors, following the instructions of the manufacturer. Following tumor induction, mice were monitored on a daily basis for body condition (*i.e.*, muscle tone and weight) and sacrificed when their body condition score was <1.5, as per guidelines of the Institutional Animal Care and Use Committee.

### Intravesical drug treatment

For these studies we used female Adeno-Cre-injected p53^*flox/flox*^; Pten^*flox/flox*^ mice because they are amenable to intravesical treatment (22). Under anesthesia, mice were placed in supine position and the external urinary orifice cleansed with betadine. A 24G Jelco angiocatheter (~10-15 mm in a typical 20 gram female mouse) was inserted through the urethra to the bladder lumen. Irrigation with sterile PBS was performed to ensure proper placement of the catheter tip, and the remaining urine was aspirated with a 1 cc syringe.

Chemotherapy agents (50 μl) were delivered into the bladder lumen and a 5-0 silk suture was tied around the urethral meatus to prevent expulsion; the installation time was 2 hours. Agents used were as follows: Cis-Diamineplatinum (II) Dichloride (Cisplatin; #479306) was purchased from Sigma; Gemcitabine (#NC0063515) and Docetaxel (#D-1000) were purchased from LC Labs. Optimal dosages for each agent were estimated based on prior literature (18, 23-25) and then confirmed in pilot studies. Cisplatin and Gemcitabine were dissolved in sterile PBS and diluted to working concentrations of 0.5 mg/ml and 25 mg/ml, respectively. Docetaxel was reconstituted to a concentration of 12.5 mg/ml in DMSO and diluted in sterile PBS to 0.5mg/ml. Each drug was delivered 1 time per week using the following dosage schedule: Vehicle (PBS) was delivered on Monday and/or Wednesdays. (Note that the Vehicle group included mice in which DMSO was diluted into PBS; these were not appreciably different than the PBS group). Cisplatin was delivered on Monday; Docetaxel was delivered on Wednesdays; Gemcitabine was delivered on Friday. When two agents were delivered, these were given on two days, rather the same day. To control for the enhanced efficacy of the combination versus the second treatment, a cohort of the single agent mice were delivered drug at twice the dose, which was not appreciably different than the single dose.

Cohorts of mice were enrolled randomly into the various treatment arms. The size of the cohorts was determined using standard power analyses, with bootstrapping from pilot studies. In particular, based on the phenotype and response to drug treatment from pilot studies, we estimated that a minimum of 6 mice would provide statistical power for analyses; however, each of experimental groups had a minimum of 10 mice in each condition. Attrition, due either to death from tumor size or infection from the catheterization, or other was less than 10% overall; the mice reported on only included those that survived to the end of the study.

### Analyses of mouse phenotypes

Following eight cycles (8 weeks) of drug treatment, mice were sacrificed and autopsied to evaluate the overall bladder phenotype and to quantify metastatic lesions. Bladders were harvested and processed for histological analysis as described (21). Metastases to distant organs were scored by visual inspection upon sacrifice, and confirmed by histological analyses; a mouse was indicated to be positive for metastases if we observed a minimum of two overt lesions. Metastases were observed mainly in the lymph nodes, pancreas, liver, and GI tract, as we have reported previously (21). Indications for sacrifice prior to eight cycles of treatment included tumor size of 1.5 cm or greater, hematuria, or weight loss of greater than 15% of initial body weight.

Immunohistochemical staining was performed on paraffin-embedded tissues as described (26). Quantification of cellular proliferation was performed by immunostaining with Ki67 (Lieca # NCL-Ki67p) using at least three independent sections on 4 independent mice/group (26). As we have reported previously, the percentage of Ki67-positively stained cells in the bladder epithelium or tumors were in comparison to the unstained cells (26). Images were captured using a whole slide scanner (Olympus VS120-S5).

### Statistical Analysis

Statistical analysis were performed using Welch t-test and Fisher’s Exact test as appropriate. GraphPad Prism software (Version 4.0) was used for statistical analysis and to generate data plots.

## Supplementary Figures


