# Supplementary material for: Preclinical analyses of intravesical chemotherapy for prevention of bladder cancer progression
Source: Oncotarget. 2013 Feb 25;4(2):269–76. doi: 10.18632/oncotarget.852 (PMC3712572; doi:10.18632/oncotarget.852)
Supplement: Supplementary file 1 [file oncotarget-04-269-s001.pdf]

Preclinical analyses of intravesical chemotherapy for prevention of bladder cancer progression - Delto et al

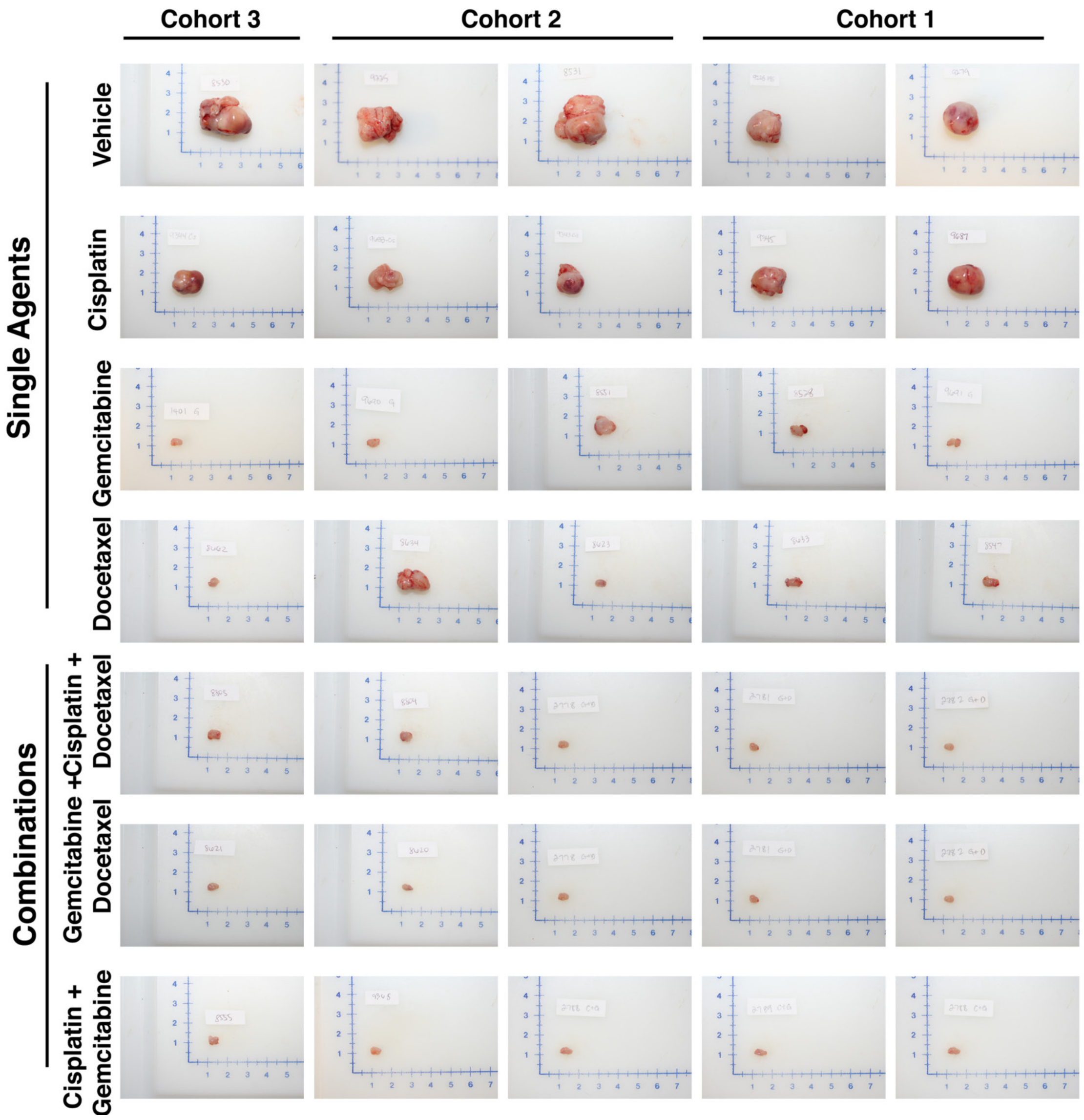

Supplementary Figure 1: Representative whole mount images of the bladder following the treatments, as indicated, and showing examples from each of three independent cohorts.
